# Overwhelming sepsis in a neonate affected by Zellweger syndrome due to a compound heterozygosis in *PEX 6* gene: a case report

**DOI:** 10.1186/s12881-020-01175-y

**Published:** 2020-11-19

**Authors:** Laura Lucaccioni, Beatrice Righi, Greta Miriam Cingolani, Licia Lugli, Elisa Della Casa, Francesco Torcetta, Lorenzo Iughetti, Alberto Berardi

**Affiliations:** 1 Neonatal Intensive Care Unit, Department of Medical and Surgical Sciences of the Mothers, Children and Adults, University of Modena and Reggio Emilia, via del Pozzo 71, 41124 Modena, Italy; 2 Post Graduate School of Paediatrics, Department of Medical and Surgical Sciences of the Mothers, Children and Adults, University of Modena and Reggio Emilia, via del Pozzo 71, 41124 Modena, Italy; 3 Pediatric Unit, Department of Medical and Surgical Sciences of the Mothers, Children and Adults, University of Modena and Reggio Emilia, via del Pozzo 71, 41124 Modena, Italy

**Keywords:** Peroxisome biogenesis disorders, Zellweger syndrome, Very long chain fatty acids, Innate immunity

## Abstract

**Background:**

Peroxisome biogenesis disorders (PBDs) are a group of metabolic diseases caused by dysfunction of peroxisomes. Different forms of PBDs are described; the most severe one is the Zellweger syndrome (ZS). We report on an unusual presentation of Zellweger syndrome manifesting in a newborn with severe and fulminant sepsis, causing death during the neonatal period.

**Case presentation:**

A term male Caucasian neonate presented at birth with hypotonia and poor feeding associated with dysmorphic craniofacial features and skeletal abnormalities. Blood tests showed progressive leukopenia; ultrasounds revealed cerebral and renal abnormalities. He died on the fourth day of life because of an irreversible Gram-negative sepsis. Post-mortem tests on blood and urine samples showed biochemical alterations suggestive of ZS confirmed by genetic test.

**Conclusions:**

ZS is an early and severe forms of PBDs. Peroxisomes are known to be involved in lipid metabolism, but recent studies suggest their fundamental role in modulating immune response and inflammation. In case of clinical suspicion of ZS it is important to focus the attention on the prevention and management of infections that can rapidly progress to death.

## Background

Peroxisome biogenesis disorders (PBDs – MIM#601539) are rare autosomal recessive disorders, caused by mutations in any of the 14 different *PEX* genes which code for peroxins, proteins involved in peroxisome assembly, including proteins involved in the import of peroxisomal matrix and membrane proteins [1].

PBDs are divided into two groups: Zellweger spectrum disorder (PBD-ZSD) and rhizomelic chondrodysplasia punctata type 1. PBD-ZSD includes three different syndromes: Zellweger syndrome (ZS - MIM# 214100), neonatal adrenoleukodystrophy (NALD - MIM# 202370) and infantile Refsum disease (IRD - MIM# 266510) ranging from severe, intermediate and mild phenotype, respectively [2–4].

PBDs prevalence is estimated to be 1 in 50.000 births in North America with huge differences worldwide. A lower incidence of ZS was reported in Japan (1 in 500.000 newborns), whereas the highest incidence was described in the Saguenay-Lac St Jean, region of Quebec (around 1 in 12.000 births) [1, 5, 6].

ZS is the most severe form of PBD-ZSD, resulting from the complete absence of functional peroxisomes. It is called ‘cerebrohepatorenal syndrome’ because of its typical clinical presentation during infancy with cerebral dysgenesis, hepatic dysfunction and evidence of renal disease. Moreover, these patients may present with typical dysmorphic features, severe hypotonia, seizures, failure to thrive and skeletal defects [5–7].

ZS can be diagnosed biochemically, detecting elevated very long chain fatty acids (VLCFA) in a fasting plasma sample, in particular elevated levels of C26:0, C26:1, ratio C24:0/C22:0, ratio C26:0/C22:0 [1, 5].

Recent reviews underline the involvement of peroxisomes in immune response and inflammation resulting in an inadequate defense in case of infection, but further studies are still required to improve knowledge about the relation between peroxisomal disorders and immunity [8–10].

We present a case of ZS in a newborn dead by a Gram-negative fulminant sepsis, whose diagnosis was confirmed only post mortem.

## Case presentation

The proband is the first child of unrelated healthy Caucasian parents. Prenatal ultrasound was uncomplicated, family history was silent.

The male neonate was born at 37 weeks’ gestation after planned caesarean section (with intact membrane and no active labor) because of breech presentation. GBS antenatal screening was negative. A placental surface culture was sterile and histological analysis of the placenta did not show chorioamnionitis. Apgar score at birth was 6 and 9 at 1st and 5th minute, respectively, body weight was 2460 g (1st centile according to Italian neonatal anthropometric charts) length was 47 cm (4th centile), while head circumference was within the normal range (33 cm, 10th centile) [11].

**Fig. 1:**
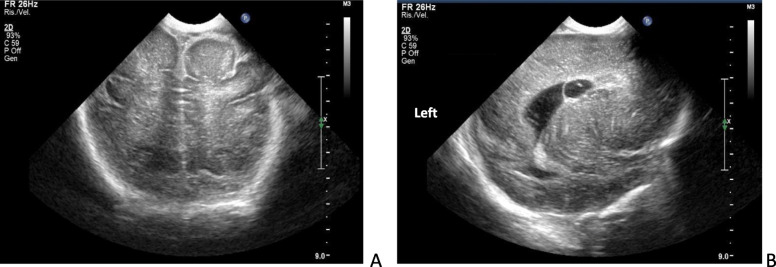
Cerebral Ultrasound images A: coronal posterior scan shows hyperechoic subcortical white matter; B: lateral sagittal scan shows ventricular pseudocysts and linear hyperechoic striae in basal ganglia

At birth the newborn presented with hypotonia, bilateral club feet and dysmorphic features (flattened facies, broad nasal bridge, micrognathia, thin lips). The baby was unable to suck but breast milk was given via orogastric tube. Glucose infusion was administered for transient hypoglycemia. Neutrophil count was at the lower limit for age (3242/mm^3^), whereas total white cell count (5.810/mm^3^), C reactive protein (0.8 mg/dl) and blood gas analysis were within a normal range [12]. Cerebral ultrasound showed mild dilated lateral ventricles with bilateral ventricular pseudocysts, hypoplasia of corpus callosum, poor insular gyration, hyperechoic subcortical white matter, and hyperechoic linear spots in the basal ganglia (Fig. 1). Abdominal ultrasound revealed bilateral renal cysts. Ophthalmologic evaluation was unremarkable. The newborn was well-appearing, except for hypotonia. At 70 h of life clinical conditions worsened rapidly. The newborn presented with respiratory failure, low blood pressure, severe metabolic acidosis, low total white cell (2400/mm^3^) and neutrophil (480/mm^3^) counts; *E. coli* was yielded from blood culture. Broad spectrum antibiotics (penicillin and gentamicin) were promptly administered. The neonate underwent mechanical ventilation and was treated with intravenous fluids, catecholamine support (dobutamine 6 μg/Kg/min; dopamine 4 μg/kg/min; adrenaline 0.02 μg/Kg/min and bolus of 0.09 mg) and *i.v.* sodium bicarbonate (8.4%, 11 ml). Despite intensive care, the baby died after 5 h.

On the basis of clinical phenotype the hypothesis of PBDs was considered. Very long chain fatty acid analysis showed C26:0 high blood levels (5.3 μmol/l; normal range 0.2–0.9 μmol/l) with elevated C24:0/C22:0 ratio (2; normal range 0.6–1.2).

A compound heterozygous mutation of *PEX6* gene was found confirming the diagnosis of Zellweger Syndrome: c.1958C > G (p.Ser653*) variant was inherited by the mother and c.2440C > T (p.Arg814*) variant was inherited by the father.

## Discussion and conclusions

Peroxisomes are small cellular organelles bound by a single membrane, containing variable compositions of proteins with detoxify functions The *PEX* genes encode for several proteins called peroxins that are involved in various stages of peroxisomal protein import and/or in the biogenesis of peroxisomes. In particular, peroxins are part of the formation of peroxisomal membranes, peroxisomal growth, fission and proliferation, and import of matrix proteins [13]. All of the approximately 46 proteins contained in peroxisomal matrix, in fact, are imported from the cytosol by a unique mechanism that does not require the imported proteins to be unfolded as they cross the membrane. In particular, *PEX6* gene, the one found mutated in our patient*,* encode for a 104 kDa peroxin that is a cytosolic AAA protein (ATPase associated with diverse cellular activities), part of PEX1–PEX6–PEX26 export complex [13]. The AAA family proteins are a group of proteins that use the energy of ATP hydrolysis to remodel molecular complexes. *PEX6* and *PEX1* form a hetero-hexameric ring, best described as a trimer of *PEX1/PEX6* dimers fundamental to import peroxisomal matrix proteins and/or vesicles fusion [14, 15]. So far, 77 different mutations in *PEX6* gene have been identified, being the second most recurrent *PEX* gene involved in PBD-ZSD genesis [16].

We describe an unusual presentation of Zellweger syndrome manifesting in a newborn with an overwhelming sepsis. Zellweger syndrome was confirmed by two pathogenic *PEX6* gene mutations, inherited from parents and combined in an compound heterozygosis previously unreported.

Although symptoms developed close to 72 h of life (that is the usual cut-off to define early- and late-onset sepsis) many data strongly support a horizontal transmission of E coli (i.e. the delivery after planned cesarean section, the negative maternal prenatal screening, the sterile placental surface cultures, the lack of evidence of chorioamnionitis at placental histological analysis, and a very long [70 h] interval free of evident symptoms of sepsis).

Moreover, poor feeding and hypotonia (2 symptoms that could be consistent with sepsis) may be more easily explained by the underlying Zellweger disease.

Zellweger disease was likely responsible for the overwhelming course of sepsis and death within a few hours. Indeed, a pivotal role of peroxisomes in regulating inflammation and antimicrobial responses has been recently hypothesized. Three main reasons support this assumption: 1) peroxisomes can contribute and modulate the cellular redox status, fundamental for the antiviral interferon-mediated cellular response, both producing and cleaning up reactive oxygen (ROS) and reactive nitrogen species (RNS) [17]; 2) peroxisomes are involved in the degradation of prostaglandins and leukotrienes, key modulators of inflammation [18, 19]; 3) peroxisomes are involved in polyunsaturated fatty acid metabolism, the backbone of a series of mediators for the resolution of inflammation such as resolvins, maresins, and protectins [20]. Peroxisomes are involved in multiple processes, such as the signaling between cells and the immune pathways, and may influence the production of inflammatory regulators as cytokines and antimicrobial peptides [7]. Finally, peroxisomes are involved in phagocytic processes, participating in tissue remodeling and maintenance of overall homeostasis. The effect of their impairment can be particularly dangerous in the central nervous system, where cells phagocytosis is essential for the elimination of excessive unwanted synapses and for the removal of overproduced apoptotic neurons and oligodendrocyte progenitor. Peroxisomes closely cooperate with mitochondria, influencing energy metabolism and lipid catabolism [7].

Thus, it is not surprising that considerable experimental evidence has recently emerged to support the concept that peroxisomes are pivotal organelles in coordinating cellular and systemic immune response strategies by serving as signaling platforms to initiate immune pathways, and by controlling the synthesis and breakdown of immune bioactive metabolites [8].

Patients with ZS have poor prognosis (often within the first year of life) due to infectious respiratory morbidity, or to severe epilepsy [1].

Increased susceptibility to infections has been also recently reported in one case of ZS. Cardoso et al. described a 2-month-old infant admitted for severe failure to thrive, with several recurrent opportunistic infections during the hospital stay, lymphopenia and thymic atrophy. Diagnosis of ZS was confirmed and a primary immunodeficiency was suspected, but subsequently it was not confirmed [21].

Due to the fulminant course of sepsis, we were unable to study the immune function, although an increased susceptibility to infections is common in many metabolic diseases. Sepsis is a life threatening condition resulting from a dysregulated response to infections. Classically, the acute phase of sepsis is characterized by an initial strong pro-inflammatory and innate immune status aimed to eliminate the pathogen, while in the later phase of sepsis there is a shift toward an anti-inflammatory and immunosuppressive status, resulting in diminished inflammation and initiation of tissue repair. Sepsis has always been considered as an inflammatory disease but recent research suggests an important contribution of other mechanisms such as coagulation, complement activation, microbiome composition, thermoregulation, circadian rhythm and metabolism. The pathogenesis of sepsis is characterized by profound changes in metabolic homeostasis and energy balance, (especially due to the failure of mitochondrial activity) [21, 22]. Therefore sepsis can overlap to metabolic disorders, worsening their clinical course.

In conclusion, ZS is a severe form of PBDs with poor prognosis. Peroxisomes are organelles involved in metabolic processes, and their role in modulating immune response and inflammation is increasingly recognized. In the clinical suspicion of ZS it is important to address promptly the diagnosis and to provide supportive therapy, particularly for preventing severe and potentially fatal neonatal infections. On the other hand, we recommend to investigate for an underlying metabolic disease in every neonate with multiple dysmorphic features and rapid worsening of suspected sepsis.

## Data Availability

All data generated or analyzed during this study are included in this published article. The data supporting this clinical case description are from previously reported studies and datasets, which have been cited. The processed data are available from the corresponding author upon request.

## Abbreviations

AAA: ATPases Associated with diverse cellular Activities
IRD: Infantile Refsum disease
NALD: Neonatal adrenoleukodystrophy
PBDs: Peroxisome biogenesis disorders
PBD-ZSD: Zellweger spectrum disorder
PMN: Polymorphonucleates
ROS: Reactive oxygen species
RNS: Reactive nitrogen species
VLCFA: Very long chain fatty acids
ZS: Zellweger syndrome
